# Lupeol Treatment Attenuates Activation of Glial Cells and Oxidative-Stress-Mediated Neuropathology in Mouse Model of Traumatic Brain Injury

**DOI:** 10.3390/ijms23116086

**Published:** 2022-05-29

**Authors:** Riaz Ahmad, Amjad Khan, Inayat Ur Rehman, Hyeon Jin Lee, Ibrahim Khan, Myeong Ok Kim

**Affiliations:** 1 Division of Life Sciences and Applied Life Science (BK 21 FOUR), College of Natural Science, Gyeongsang National University, Jinju 52828, Korea; riazk0499@gnu.ac.kr (R.A.); amjadkhan@gnu.ac.kr (A.K.); inayaturrehman201516@gnu.ac.kr (I.U.R.); lhj4912@gnu.ac.kr (H.J.L.); ibrahim1994@gnu.ac.kr (I.K.); 2 Alz-Dementia Korea Co., Jinju 52828, Korea

**Keywords:** lupeol, traumatic brain injury, glia activation, neuroinflammation, oxidative stress, cognitive impairment, neurodegenerative diseases

## Abstract

Traumatic brain injury (TBI) signifies a major cause of death and disability. TBI causes central nervous system (CNS) damage under a variety of mechanisms, including protein aggregation, mitochondrial dysfunction, oxidative stress, and neuroinflammation. Astrocytes and microglia, cells of the CNS, are considered the key players in initiating an inflammatory response after injury. Several evidence suggests that activation of astrocytes/microglia and ROS/LPO have the potential to cause more harmful effects in the pathological processes following traumatic brain injury (TBI). Previous studies have established that lupeol provides neuroprotection through modulation of inflammation, oxidative stress, and apoptosis in Aβ and LPS model and neurodegenerative disease. However, the effects of lupeol on apoptosis caused by inflammation and oxidative stress in TBI have not yet been investigated. Therefore, we explored the role of Lupeol on antiapoptosis, anti-inflammatory, and antioxidative stress and its potential mechanism following TBI. In these experiments, adult male mice were randomly divided into four groups: control, TBI, TBI+ Lupeol, and Sham group. Western blotting, immunofluorescence staining, and ROS/LPO assays were performed to investigate the role of lupeol against neuroinflammation, oxidative stress, and apoptosis. Lupeol treatment reversed TBI-induced behavioral and memory disturbances. Lupeol attenuated TBI-induced generation of reactive oxygen species/lipid per oxidation (ROS/LPO) and improved the antioxidant protein level, such as nuclear factor erythroid 2-related factor 2 (Nrf2) and heme-oxygenase 1 (HO-1) in the mouse brain. Similarly, our results indicated that lupeol treatment inhibited glial cell activation, p-NF-κB, and downstream signaling molecules, such as TNF-α, COX-2, and IL-1β, in the mouse cortex and hippocampus. Moreover, lupeol treatment also inhibited mitochondrial apoptotic signaling molecules, such as caspase-3, Bax, cytochrome-C, and reversed deregulated Bcl2 in TBI-treated mice. Overall, our study demonstrated that lupeol inhibits the activation of astrocytes/microglia and ROS/LPO that lead to oxidative stress, neuroinflammation, and apoptosis followed by TBI.

## 1. Introduction

Brain damage is serious global health problem and is the chief cause of death and disability [1,2], connected to dementia and chronic neurodegeneration [3,4]. Traumatic brain injuries (TBIs) can range from mild to severe, and most TBI victims suffer from mild impairment characterized by short-term and long-term physical, emotional, behavioral, and cognitive impairments from brain damage [5]. The mechanism of TBI is a complex pathological process consisting of two-step process [6,7]. The first step is mechanical injury to brain tissue that directly leads to neuronal damage and blood vessel destruction [8,9,10,11,12,13], the second step involves the spread and weakening of damage to the surrounding microenvironment due to several factors such as proinflammatory response, oxidative stress, BBB dysfunction, apoptosis, and neurotoxic accumulation, all occurring immediately after the primary injury [14,15,16,17,18,19].

TBI is associated with post-traumatic stress disorder and chronic neuroinflammation [20], which is strongly associated with microglia activation and is characterized by a variety of indications, including the synthesis and secretion of proinflammatory cytokines and chemokines [21,22,23], that can become neurotoxic and further contribute to neurodegenerative diseases, such as Alzheimer’s disease, Parkinson’s disease, multiple sclerosis, and amyotrophic lateral sclerosis [21,22,24,25,26]. Mitochondrial dysfunction and reactive oxygen species (ROS) generation, due to direct or indirect damage after TBI, have also been identified as triggers of neuroinflammation [27]. ROS plays an important role as a secondary messenger in many intracellular signaling pathways and also as a mediator of oxidative stress and inflammation [28]. Increasing evidence suggests that ROS-induced oxidative stress may play an important role in the pathophysiology of secondary injury after TBI [29,30]. ROS also reacts with polyunsaturated fatty acids in the lipid membranes causing lipid peroxidation (LPO) [31].

It is believed that TBI generates a variety of events and is a threat factor to the pathogenesis of various neurodegenerative diseases, such as Alzheimer’s disease (AD) [32] and Parkinson’s disease (PD) [33]. Neuroinflammation and oxidative stress are the key features of neuropsychiatric disorders and outcomes after brain injury [34]. TBI initiates a chronic biochemical process leading to microglia/astrocytes activation and neuritis that may contribute to later neuronal dysfunction [25,35,36]. Several studies have publicized that microglia release proinflammatory mediators that contribute to neuronal dysfunction and cell death in response to injury and various immune stimuli [24,37,38]. Activated microglia also release several neurotoxic substances, such as reactive oxygen and nitrogen species, that can exacerbate neuronal death [39].

In addition to neuroinflammation, oxidative stress has also been well known to induce neuroinflammation in TBI. Once injury-induced excitotoxicity occurs, the ROS and nitric oxide (NO) production is increased, where protective mechanisms such as antioxidants fail to control these radicals, leading to oxidative stress [40]. Nuclear factor erythroid-2-related factor 2 (Nrf2) is a basic leucine zipper redox-sensitive transcription factor that regulates the redox state of cell under harmful stresses [41]. Under normal conditions, Nrf2 is localized in the cytoplasm and is attached by its inhibitor, Kelch-like ECH-associated protein 1 (Keap1). Under conditions of oxidative stress, Nrf2 dissociates from Keap1, translocates to the nucleus, and regulates antioxidant genes expression, such as heme oxygenase-1 (HO-1) [42,43,44]; however, increased oxidative stress is involved in deregulation of Nrf2/HO-1 signaling and the endogenous antioxidant system [45]. Furthermore, the increased levels of oxidative stress and neuroinflammation disrupt the proper structure and function of neurons, as well as synaptic, memory, and cognitive functions in the brain [46,47]. Various studies have shown that activated microglia, astrocytes, and raised ROS mediate phosphorylated-nuclear factor kappa B (p-NF-κB) and also upregulates other inflammatory mediators, such as TNF-α, COX-2, and interleukins, which further decline the neuronal structure and function [23,48,49,50,51,52,53,54,55]. Moreover, TBI can also induce apoptotic proteins, such as caspase-3, Bax, and cytochrome-C, while reducing regulated protein Bcl2 [56].

Despite new understanding of the pathological process of TBI, effective treatment strategies have not yet emerged. In today’s context, there is no supportable treatment for brain-damage-caused neuropathology due to lack of adequate knowledge and the failure to develop drugs due to several pathological conditions in the brain [57]. To create more effective neuroprotective treatments for TBI it is necessary to understand the complex cellular and molecular events contributing to brain injury [58]. Many medicinal plants and their secondary metabolites are reported to have the ability to improve the symptoms of neurodevelopmental disorders [59]. Natural compounds have potential to target multiple components of the brain injury [60]. Lupeol is a triterpenoid compound found in vegetables, fruits, and several medicinal plants [61,62], and has an extensive range of biological effects, such as anticancer [63], antidiabetic, antimicrobial, and hepatoprotective effects [64]. It has also been shown to exhibit antioxidant and anti-inflammatory effects [65,66]. Lupeol provides a broad range of neuroprotective effects against several stimuli, such as oxidative stress, free radical generation, and neuroinflammation, by suppressing activated glial cells and inflammatory mediators, and by reducing Aβ accumulation in mouse brain [67,68]. Several studies have shown that lupeol has antiapoptotic properties, which include inhibition of the mitochondrial apoptotic pathway and ROS production [69,70].

The current study was designed to evaluate the neuroprotective role of lupeol in experimental TBI. We developed a cortical stab wound injury in a mouse model that showed oxidative stress, glial-cells-activation-mediated neuroinflammation, and apoptosis. The mouse model was treated using lupeol to explore its therapeutic potential in brain injury. Administration of lupeol ameliorated oxidative stress, glial-cells-activation-mediated neuroinflammation, and apoptosis-induced neurodegeneration. Taken together, the data showed that administration of lupeol recompenses neuropathology in an animal model of brain injury.

## 2. Results

### 2.1. Lupeol Treatment Inhibits Glial Cell Activation in the Cortex and Hippocampus of Mouse Brain with TBI

**Figure 1:**
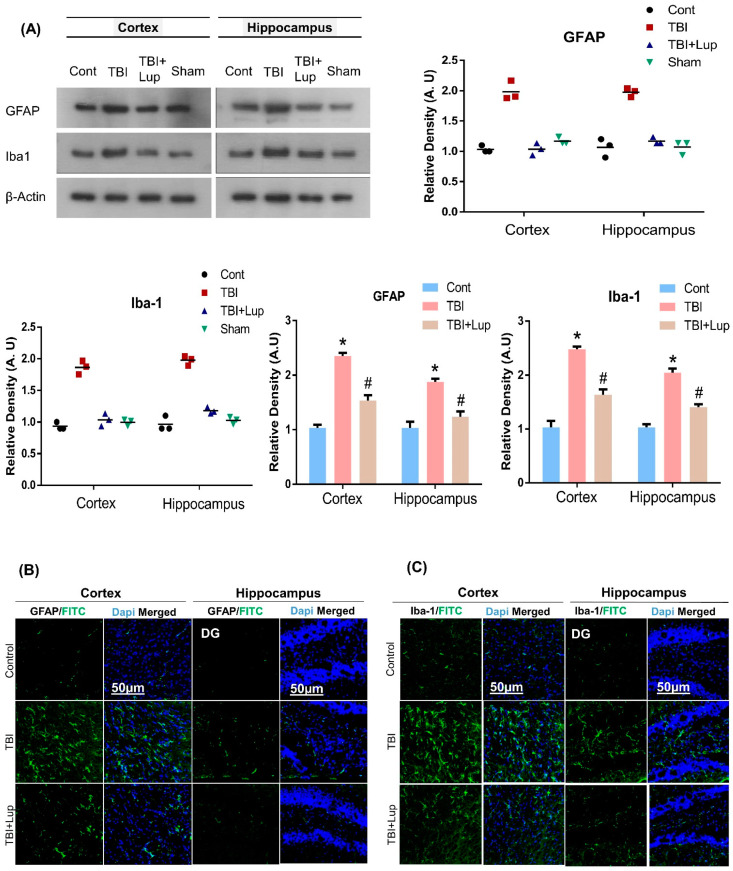
Lupeol ameliorates TBI-induced microglia/astrocytes activation in mouse brain: (**A**) Western blot analysis showing the expression of GFAP and Iba-1 in the experimental mice (cortex and hippocampus regions); (**B**,**C**) confocal photomicrographs showing the immunoreactivity of GFAP and Iba-1 (FITC—green), stained with DAPI (blue) in the cortex and DG region of hippocampus in different mice groups. The cropped bands were quantified using Image software, and the differences are represented in the histogram. Asterisk (*) sign indicates significant difference from the normal saline-treated group; hash (#) sign indicates significant difference from the TBI-treated group. Bar = 50µm. The density values are expressed in arbitrary units as the mean ± SEM of the indicated proteins (*n* = 6 animals per group). Significance = *p* ≤ 0.05.

Microglia and Astrocytes play important roles in brain development by regulating neuronal number and synapse formation. Traumatic brain injury induces structural and functional alterations of astrocytes and microglia [71,72,73]. It has been reported that early microglia and astrocytes activation following traumatic brain injury (TBI) may contribute to the restoration of homeostasis in the brain [73,74,75]. To determine whether lupeol could reverse TBI-induced activation of astrocytes and microglia in mouse brain, we carried out Western blot and immunofluorescence analysis. Our immunoblot results specified a significant increase in the protein expression of GFAP and Iba-1 in the cortex and hippocampus of TBI-alone group compared with those of saline-treated (control) mice (Figure 1A). However, TBI + Lupeol co-treatment significantly reduced the elevated expression of GFAP as compared with the TBI-alone group. For further confirmation, the GFAP and Iba-1 expression were also investigated using immunofluorescence staining. In accordance with the immunoblot results, the immunofluorescence analysis also suggested that immunoreactivity of GFAP and Iba-1 was significantly increased in TBI-alone group as compared with the saline-treated (control) group (Figure 1B,C). Interestingly, the TBI + Lupeol co-treatment significantly reduced the immunofluorescence reactivity of GFAP and Iba-1 as compared with the TBI-alone group. These findings indicate that lupeol can inhibit the activation of glial cells in mouse brain.

### 2.2. Lupeol Treatment Induces Nrf2/HO-1 Expression and Attenuates ROS and LPO in Mouse Brain with TBI

**Figure 2:**
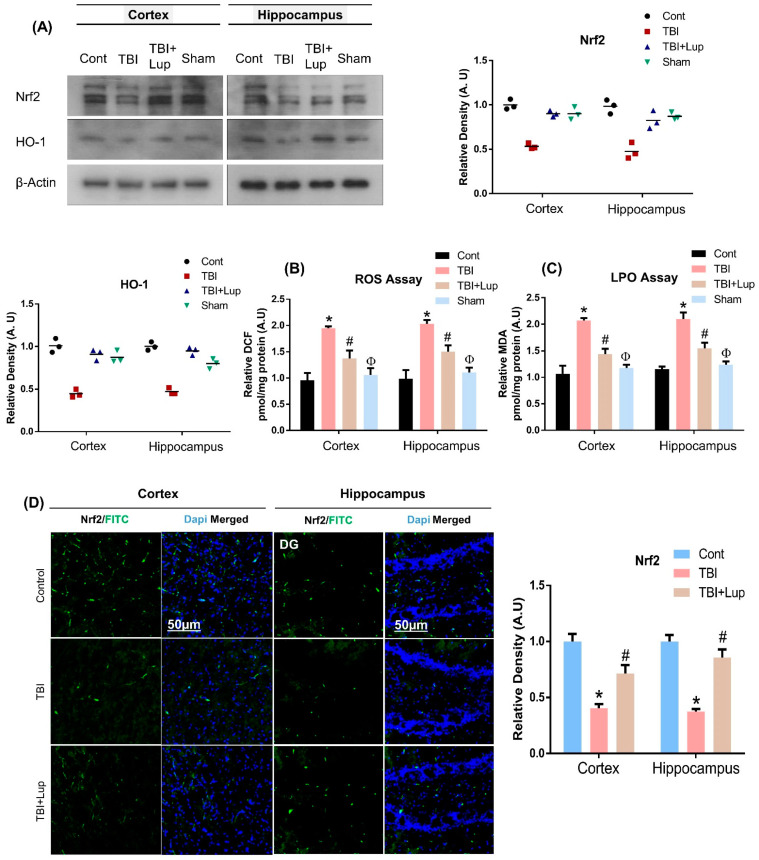
Lupeol treatment inhibited TBI-induced oxidative stress and ameliorated ROS/LPO production in the cortical and hippocampal region of mouse brains: (**A**) Western blot analysis representing the expression of Nrf2 and HO-1 in the cortex and hippocampus of mouse brain; (**B**,**C**) the analysis of the generation of ROS and LPO production in vivo in the mouse brain (cortex and hippocampus); (**D**) immunofluorescence analysis of Nrf2 immunoreactivity (FITC—green), stained with DAPI (blue) in the cortex and DG region of hippocampus in different experimental mice groups. The cropped bands were quantified using Image software, and the differences are represented in the histogram. Asterisk (*) sign indicates significant difference from the normal saline-treated group; hash (#) sign indicates significant difference from the TBI-treated group, while the phi (Φ) sign indicates no significant from normal saline-treated control group. Bar = 50 µm. The density values are expressed in arbitrary units as the mean ± SEM of the indicated proteins (*n* = 6 animals per group). Significance = *p* ≤ 0.05.

Several studies have reported that the brain is susceptible to oxidative stress [76]. Oxidative stress has been well documented in TBI. Once injury-induced excitotoxicity occurs, the production of ROS and nitric oxide (NO) is increased, where protective mechanisms such as antioxidants fail to control these radicals, leading to oxidative stress [40]. To explore the antioxidant potential of Lupeol against TBI–induced oxidative stress, we conducted ROS and LPO assays. Our results indicated elevated ROS levels and increased DCF contents in TBI mouse brains as compared with the saline-treated (control) mouse brain, which were significantly reduced by lupeol treatment (Figure 2B). Similarly, there was an enhanced expression of LPO (MDA level) in the TBI-alone group, which was significantly attenuated with lupeol treatment (Figure 2C). Moreover, the antioxidant potential of Lupeol was further evaluated by analyzing the protein expression of Nrf2 and HO-1 in the mouse brain. Our results indicated a reduced level of Nrf2 and HO-1 in TBI-treated mouse brain, but the TBI + Lupeol co-treatment group significantly reversed the Nrf2 and HO-1 level in both cortex and hippocampus the mouse brain (Figure 2A). We further confirmed our results through immunofluorescence analysis that indicated a decreased expression level of Nrf2 in the cortex and hippocampal region of the TBI mouse group, while the level was significantly raised in the TBI + Lupeol co-treatment mouse group (Figure 2D), suggesting that antioxidant lupeol treatment mitigates oxidative stress induced by TBI in mouse brain.

### 2.3. Administration of Lupeol Reduced the Expression of Inflammatory Cytokines in Mouse Brain with TBI

**Figure 3:**
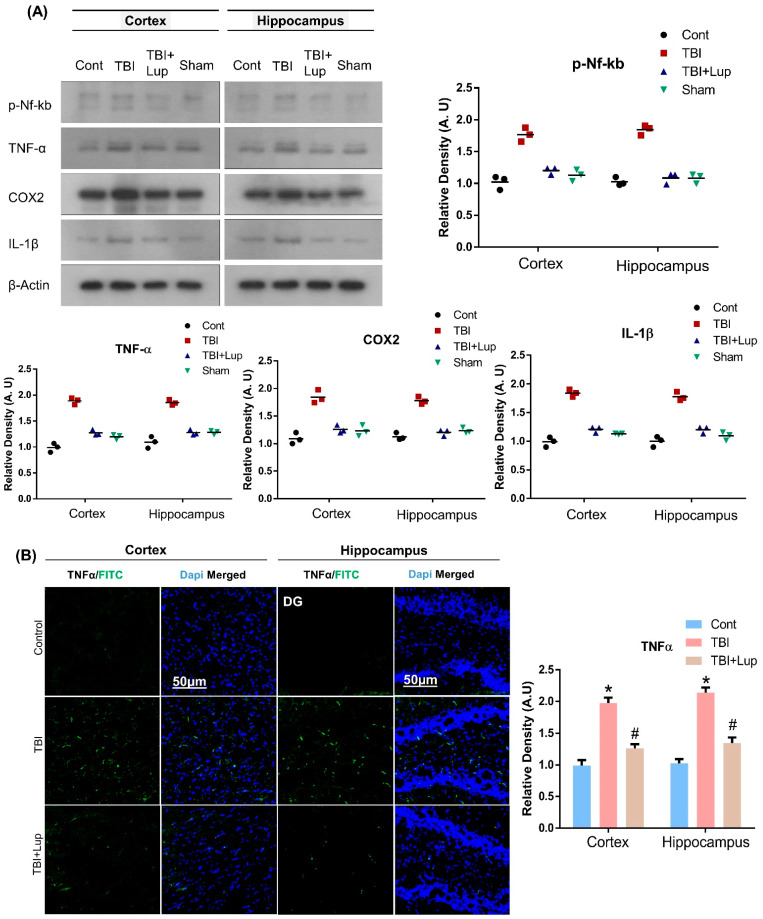
Lupeol alleviates the expression of p-NF-kB and inflammatory cytokines in TBI-treated mouse brain: (**A**) Western blot analysis of p-NF-kB, TNF-α, COX2, and IL-1β expression in the cortex and hippocampus of mouse; (**B**) immunofluorescence analysis of TNF-α immunoreactivity (FITC—green), stained with DAPI (blue) in the cortex and DG region of hippocampus in different experimental mice groups. The cropped bands were quantified using Image software, and the differences are represented in the histogram. Asterisk (*) sign indicates significant difference from the normal saline-treated group; hash (#) sign indicates significant difference from the TBI-treated group. Bar = 50µm. The density values are expressed in arbitrary units as the mean ± SEM of the indicated proteins (*n* = 6 animals per group). Significance = *p* ≤ 0.05.

In response to TBI, glia activation and oxidative stress leads to neuroinflammation [77,78,79]. In the central nervous system, a variety of stimuli, such as injury, oxidative stress, and neurotoxins induce p-NF-κB, which upregulates other inflammatory cytokines such as tumor necrosis factor alpha (TNFα), cyclooxygenase-2 (COX2), and interleukin 1 beta (IL-1β) [80,81]. In order to examine the effects of lupeol on TBI-mediated neuroinflammation, we conducted immunoblot and immunofluorescence analyses. Our immunoblot results exposed that, compared with the saline-treatment (control) group of mice, the TBI-alone mice group showed significantly increased protein expression level of p-NF-κB and its associated downstream neuroinflammatory mediators, including TNFα, COX-2, and IL-1β in the cortex and hippocampus regions (Figure 3A). Interestingly, TBI + Lupeol co-treatment significantly reduced the elevated expression of p-NF-κB and its related downstream inflammatory mediators. To further strengthen these results, we also performed confocal microscopy. In accordance with our immunoblot results, the immunofluorescence analysis also suggested that TNFα immunoreactivity significantly increased in the TBI-alone group as compared with the normal saline-treated (control) mice group. Interestingly, TBI + Lupeol co-treatment significantly reduced immunofluorescence reactivity of TNFα in both the cortex and hippocampus regions (Figure 3B). Taken together, these results indicate that lupeol is effective against TBI-induced neuroinflammation in the mouse brain.

### 2.4. Lupeol Treatment Attenuated TBI-Induced Apoptotic Cell Death in Mouse Brain

**Figure 4:**
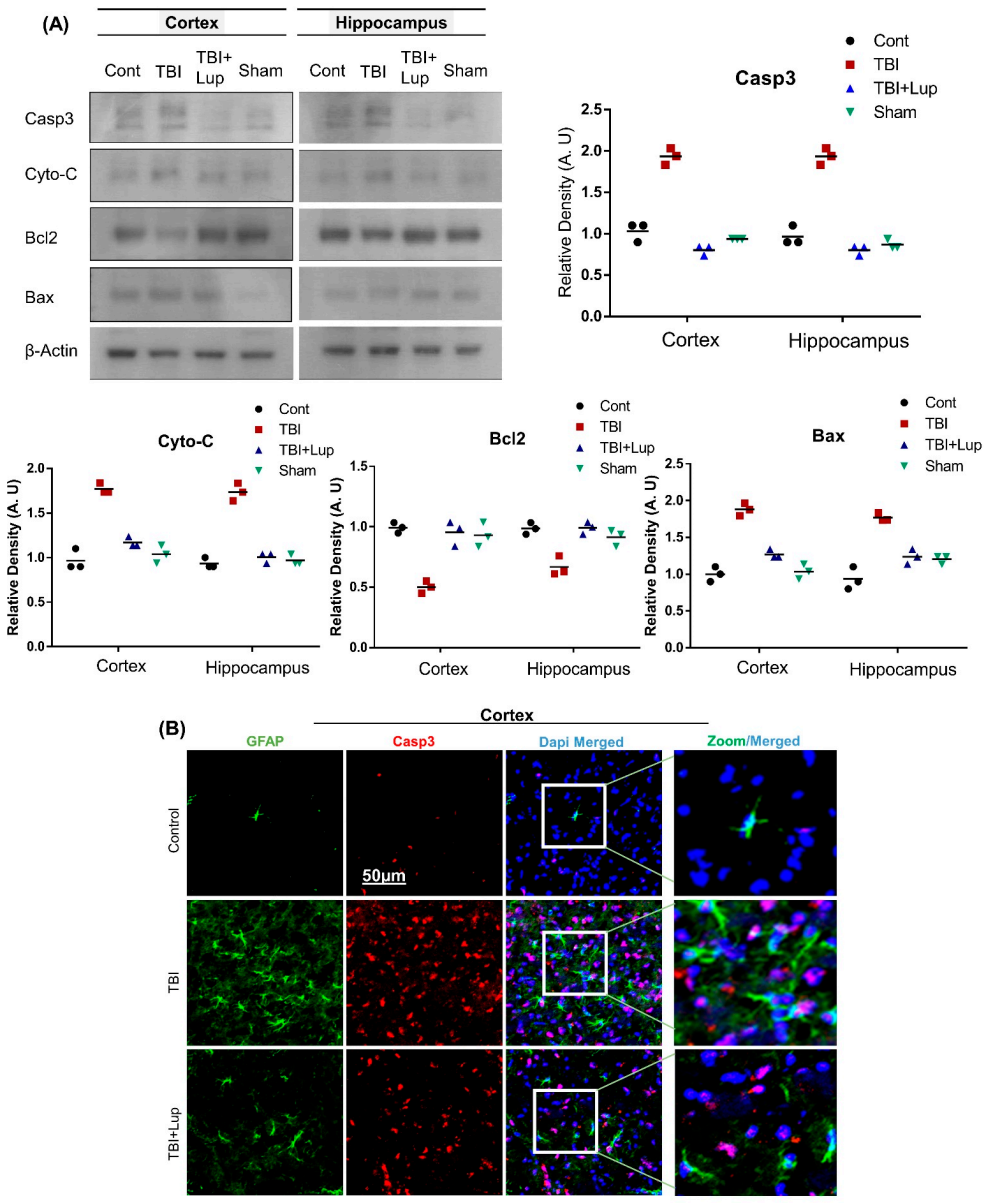

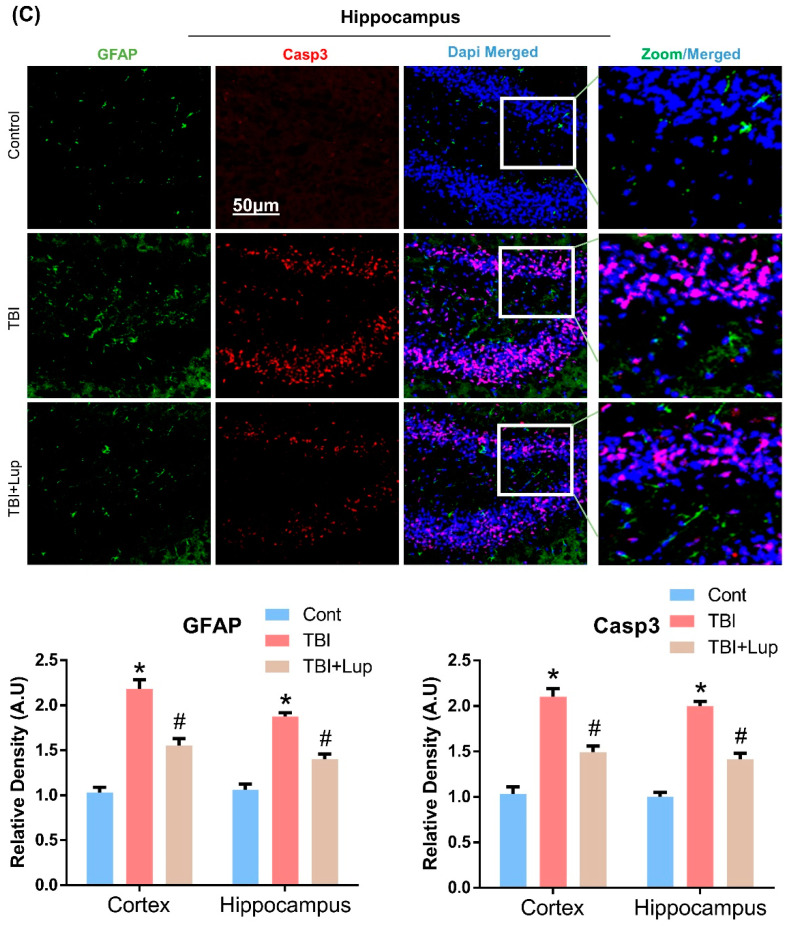
Lupeol reversed the TBI-induced apoptotic cell death in the mouse brain. (**A**) Western blot analysis of proapoptotic protein caspase-3, Bax, cytochrome-C, and antiapoptotic protein expression (Bcl2) in the cortex and hippocampus of mice; (**B**) immunofluorescence images of the colocalized reactivity of GFAP (FITC—green), Casp3 (red), stained with DAPI (blue) in the cortex region; (**C**) immunofluorescence images of the colocalized reactivity of GFAP (FITC—green), Casp3 (red), stained with DAPI (blue) in the hippocampus region. The zoom represents high magnification. The cropped bands were quantified using Image software, and the differences are represented in the histogram. Asterisk (*) sign indicates significant difference from the normal saline-treated group; hash (#) sign indicates significant difference from the TBI-treated group. Bar = 50µm. The density values are expressed in arbitrary units as the mean ± SEM of the indicated proteins (*n* = 6 animals per group). Significance = *p* ≤ 0.05.

Several studies have been reported that TBI causes apoptotic cell death and neurodegeneration [53,56], which are associated with behavioral dysfunction and histopathological changes [82]. To determine the effects of lupeol against apoptotic neurodegeneration mediated by TBI, we conducted immunoblotting analysis for proapoptotic markers (caspase-3, Bax, and Cyto-C) and an antiapoptotic marker (Bcl-2) in all the experimental groups. In comparison with the saline-treated (control) group, there was an increased expression of caspase-3, Bcl-2, Bax, and Cyto-C and downregulation of Bcl-2 in the TBI-treated group of mice (Figure 4A). Interestingly the TBI + Lupeol co-treatment regulated the apoptotic signaling molecules in both cortex and hippocampus regions the mouse brain. To strengthen our immunoblot results, we also conducted double immunofluorescence analysis of caspase-3 and GFAP. The immunofluorescence analysis of caspase-3 and GFAP also indicated an elevated expression in both cortex (Figure 4B) and hippocampus (Figure 4C) of the TBI-treated group as compared with the saline-treated (control) group. However, the TBI + Lupeol co-treatment significantly decreased the expression levels of caspase-3 and GFAP (Figure 4B,C).

### 2.5. Lupeol Treatment Improved Learning Memory and Spontaneous Alteration Behavior in TBI-Induced Memory Impairment

**Figure 5:**
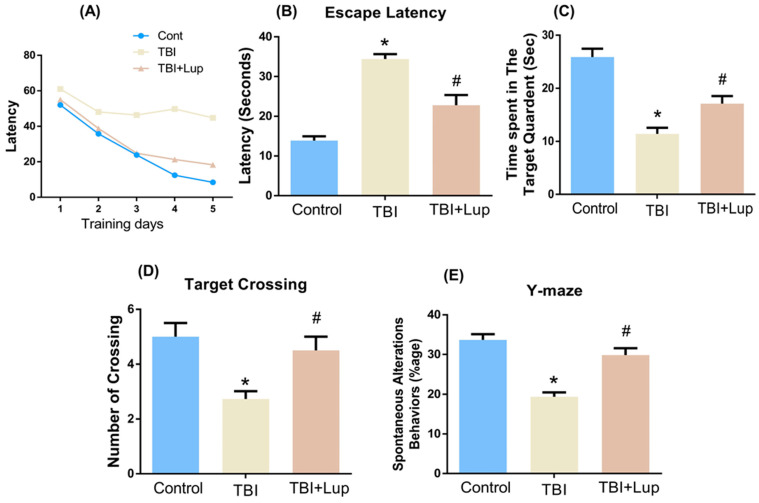
Lupeol improved memory, learning, and cognitive behavior in TBI-treated mice: (**A**) mean escape latency to reach the hidden platform during training (5 days) with its representative trajectories at day 6 and (**B**) at the 6th day after training; (**C**,**D**) the time spent in the target quadrant where the hidden platform was previously present and the number of crossings over that location in the absence of a platform; (**E**) the Y-maze analysis representing the spontaneous alteration behaviors of mice and its representative trajectories. Asterisk (*) sign indicates significant difference from the normal saline-treated group; hash (#) sign indicates significant difference from the TBI-treated group. The density values are expressed in arbitrary units as the mean ± SEM of the indicated proteins (*n* = 6 animals per group). Significance = * *p* ≤ 0.05, # *p* ≤ 0.05.

In order to analyze cognitive abnormalities in all experimental mice groups, we conducted the behavioral tests. Our tests demonstrated that the TBI-treated mice showed memory glitches, as shown in the MWM test. In the MWM test, the mean latency (to find hidden platform) slowly reduced over training days in all mouse groups, except the TBI-treated group, which showed a longer latency as compared with the saline-treated (control) group. However, in comparison with the TBI-alone group, the TBI + Lupeol co-treatment group showed significantly reduced latency in finding the platform (Figure 5A,B). After completion of the trial period, the hidden platforms were removed and the probe test was performed. We found that the number of platform crossings were significantly increased in the TBI + Lupeol co-treated group compared with the TBI-alone group (Figure 5D). Additionally, we found that TBI + Lupeol co-treated mice spent more time in the target quadrant as compared with the TBI-treated mice (Figure 5C), showing that lupeol reduced TBI-induced memory impairment. To analyze spatial working memory, we performed the Y-maze test, based on the spontaneous alteration behavior percentage (%). We found that the alternation behaviors percentage (%) was significantly reduced in the TBI-alone group as compared with the saline-treated (control) mice, indicating impaired working memory. However, the TBI + Lupeol co-treated group showed a significant increase in spontaneous alteration behavior (%) in comparison with the TBI-alone group (Figure 5E), indicating that lupeol attenuated short-term memory deficits in the TBI-treated mice.

## 3. Discussion

This study presents the role of lupeol in inhibiting the activation of astrocytes/microglia and ROS/LPO, providing neuroprotection after TBI in mice. The brain is extremely sensitive to lipid peroxidation (LPO) because of its high content of polyunsaturated fatty acids and iron [83]. ROS are byproducts of normal metabolism of oxygen which includes free radicals such as superoxide, hydroxyl radicals, and singlet oxygen [84]. Trauma induces excessive production of ROS, induced from excitotoxicity and depletion of the endogenous antioxidant system (e.g., superoxide dismutase, catalase, and glutathione peroxidase), and causes lipid peroxidation, protein oxidation, mitochondrial dysfunction, DNA cleavage, and changed signal transduction [85,86,87]. Current studies have proved that a key transcription factor, nuclear factor erythroid 2-related factor 2 (Nrf2), has an essential role in the induction of endogenous antioxidant enzymes against oxidative stress [88]. However, the impairment of Nrf2 signaling leads to oxidative stress [89]. HO-1, implicated in the endogenous defense response, represents an important cytoprotective mechanism against oxidative damage by breakdown of toxic heme into free iron, carbon monoxide, and biliverdin [90,91]. HO-1 also facilitates cellular responses to oxidative stress. In this study, we aimed to investigate the oxidative stress and cellular energy imbalance after TBI in mouse brain and the modulating effect of energy homeostasis through the antioxidant lupeol in the brain of TBI mice. Our ROS and LPO analyses clearly indicated that oxidative stress was increased in the mouse brain after TBI. In addition, the antioxidants Nrf-2 and HO-1 were shown to be downregulated via our Western blot results in the mouse brain that received TBI. However, it was interesting to find that oxidative stress levels were reversed in the mouse brains treated with lupeol, as revealed by ROS and LPO analyses and elevated expression of antioxidants Nrf-2 and HO-1. Our findings suggest that lupeol may mitigate TBI-induced oxidative stress by inducing Nrf2 and HO-1 expression.

**Figure 6:**
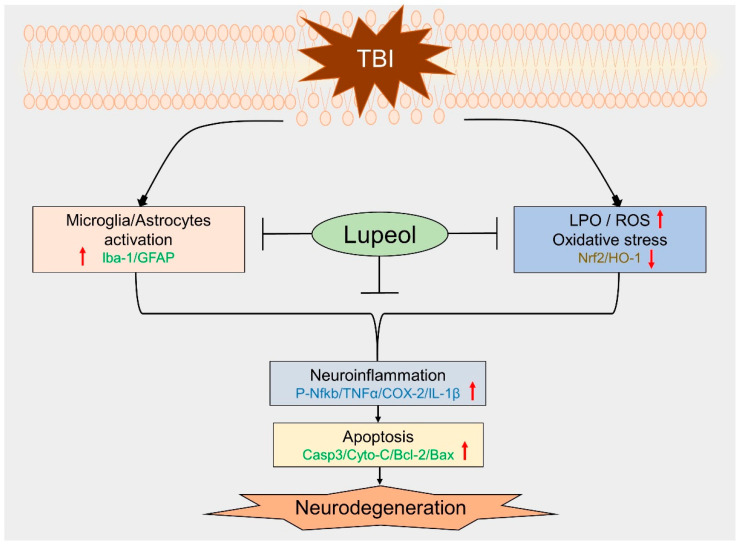
Suggested graphical representation of possible Lupeol neuroprotective effect against TBI-induced neurotoxicity. Lupeol (a triterpenoid) reduced TBI-induced neuroinflammation, oxidative stress, neuroapoptosis, memory impairment, and neurodegeneration induced by TBI in mouse brain. (Upward direction of arrow indicates activation/upregulation, while downward direction indicates downregulation).

A vital feature of TBI is the development of neuroinflammation, primarily due to glial activation [92]. Glial activation may be either advantageous or detrimental, depending on the degree of activity and longevity. Thus, the expression and release of neurotrophic or noxious substances in response to nerve damage is likely a determinant of neuronal viability [93]. An important feature of TBI is increased GFAP expression, signifying activation, and/or proliferation of astrocytes [94]. When injury occurs, microglia are rapidly activated, modifying their normal morphology to forked cell structures and larger cell bodies [95]. Early microglia activation in TBI promotes astrogliosis and persistent inflammation [96]. Astrocytes and microglia link with each other through the cytokines and other extracellular mediators they release [97,98]. They play critical roles in the brain’s ion and water homeostasis, energy metabolism, blood–brain barrier, and immune response, and they change their morphology and protein expression, releasing both pro- and anti-inflammatory mediators [99]. In the present study, our results elucidated that lupeol reduced the number of activated astrocytes and microglia in the adult mouse cortex and hippocampus regions. Activated microglia/astrocytes and oxidative stress are responsible for the release of inflammatory molecules, such as TNF-α, IL-1β, and COX-2, that cause neuroinflammation. It is broadly known that the inflammatory response of the brain after TBI contributes to widespread apoptosis and chronic tissue degeneration. Neuroinflammation is one of the important factors among the pathophysiological signs of TBI [100]. The transcription factor NF-κB is a known immunomodulatory factor in various cell types [101]. Astrocytes activation and Nrf2 downregulation by TBI also activate NF-κB [89], which mediates inflammation through upregulation of the Na/K/2Cl cotransporter (NKCC) [102]. Likewise, TNF-α, which is produced by microglia, astrocytes, and neurons [103], is an important mediator of the inflammatory response in TBI [104]. It also appears to be released earlier than other proinflammatory cytokines and initiates the activation of several cytokines, growth factors, and the recruitment of immune cells [105,106]. Moreover, another inflammatory cytokine, COX2, has been associated with worse outcomes after brain injury as well as early-onset dementia [107,108]. Elevated COX2 expression has been observed in head trauma and several progressive neurodegenerative conditions, e.g., Alzheimer’s disease and Parkinson’s disease [109,110]. Similarly, an increasing number of studies have reported that immune-reactive IL-1β cells are overexpressed in pathogenic conditions, brain injury, and degeneration. Overexpressed IL-1β disturbs both neuronal and non-neuronal cells of the CNS [111]. Moderate and severe TBI both increase IL-1β mRNA and protein levels in the cortical and deep brain structures after injury [112]. We therefore analyzed the expression levels of these inflammatory mediators through Western blot and confocal microscopy. Our findings illuminated that the TBI+ Lupeol co-treatment suppressed p-NF-κB, and its associated proinflammatory mediators (TNF-α, IL-1β, and COX-2) in the indicated region (cortex and hippocampus) of mouse brain, as described in the diagram (Figure 6). 

Proapoptotic-related proteins, such as Bax and caspase-3, can also be induced by TBI [56]. Therefore, we also investigated the protein expression of active caspase-3, which leads to cleavage of many proteins, ultimately leading to DNA fragmentation and apoptosis [113]. Activation of caspase-3 is also supported by studies demonstrating cellular modifications including neuronal cell death within the brain tissue of TBI patients [114], and has been considered a key feature of neurodegenerative diseases [115]. Caspase-dependent and caspase-independent apoptosis are both regulated by proteins of the Bcl-2 family, which include both pro-death and pro-survival members [90,116]. Bcl-2 family proteins regulate the permeability of the outer mitochondrial membrane and the formation of permeable transition pores [117]. They contain highly conserved Bcl-2 homology domains (BH 1–4), which are important for homo- and heterocomplex formations. Complexes formed between proteins containing BH-3 domains, such as Bax, can promote the release of cytochrome-*c* from mitochondria [90]. It has also been reported that, in the acute post-traumatic period, damaged neurons showed a sharp decrease in Bcl-2 expression, followed later by an increase in Bax mRNA and protein [118]. In our study, we revealed that lupeol downregulated the expression of Cyto-C, Bax, and Casp-3, while upregulating Bcl2 expression. Several recent studies have confirmed the therapeutic potential of lupeol, and it has been shown that lupeol provides neuroprotection via suppressing inflammation and apoptosis [68]. Similarly, our behavior analysis (MWM and Y-maze tests) also indicated that TBI significantly impaired cognitive and learning behavior. However, the TBI + Lupeol co-treatment considerably reversed these effects and improved cognition, spatial learning, and memory processing. Taken together, our findings prove that lupeol is effective in reversing TBI-induced memory impairment in mouse brain.

## 4. Materials and Methods

### 4.1. Animals

Male wild-type C57BL/6 N mice (8 weeks old, body weight 25–30 g, *n* = 42) were purchased from Samtako Bio (Osan, Republic of Korea). The animals were kept in Ostrich’s mouse cages and were acclimatized for 7 days; they were maintained under controlled temperature (25 °C) and lighting (12/12 dark/light cycle). Each mouse group (12 mice/group) was kept in two cages (6 mice/cage) and were allowed free access to water and food as previously described [119]. All the experimental techniques were performed according to the protocol approved by the Animal Ethics Committee of the Division of Applied Life Sciences, Gyeongsang National University, South Korea (Approval ID: 125, 3 June 2020).

### 4.2. Traumatic Brain Injury of Mice and Drug Treatment

**Figure 7:**
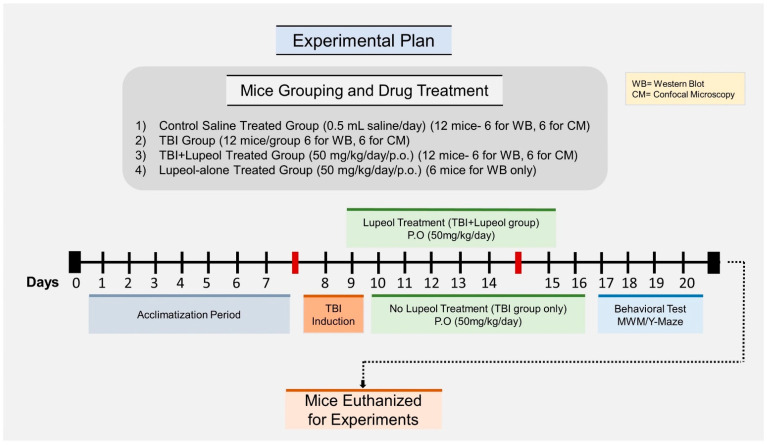
Schematic diagram of the experimental design showing the duration of the TBI and/or lupeol administration in adult mice and the behavioral analysis.

Mice were randomly arranged into four groups: (1) a control saline-treated group (control, *n* = 12), which received saline for one week as a vehicle; (2) traumatic brain injury group (TBI, *n* = 12); (3) traumatic brain injury group with lupeol treatment (TBI + Lup, *n* = 12) (lupeol dose: 50 mg/kg/day/mice/p.o.); and (4) sham-treated group (Lup, *n* = 6). The sham-treated group received a daily injection of lupeol (50 mg/kg/day/mice/p.o.) for 7 days without head injury (Figure 7). Lupeol dosages were selected according to previously published studies [67,68]. Lupeol (CAS Number*: 545-47-1) was purchased from Sigma Co. (St. Louis, MO, USA). For the surgical procedures, the mice were anesthetized with Zoletil (0.1 mL/100 g body weight) and Rompun (0.05 mL/100 g body weight) and placed on a stereotaxic frame. Surgery was performed on the animals in a temperature-controlled environment. The skull was exposed by removing the skin (mid-longitudinal incision). TBI was induced by a sharp edge scalpel blade inserted 3 mm into right hemisphere of the brain. The scalpel blade remained in the brain for 1 min and was slowly removed. The skull was covered with bone wax and the skin was closed with a silk suture. The experiments were performed under continuous heating with a heating lamp and mice were monitored visually until full recovery, as previously described [57,120].

### 4.3. Morris Water Maze (MWM) Test

For behavior analysis, The Morris water maze (MWM) test was performed as previously described [121,122]. The MWM apparatus consisted of a round tank (100 cm in diameter, 40 cm high, and 15.5 cm deep) filled with water and made opaque with white ink. A transparent platform 20 cm in height and 10 cm in diameter was hidden 1 cm below the water surface in one quadrant of the tank during the experiment. After three days, following TBI and lupeol treatment, all mice were brought into the MWM apparatus to assess the cognitive ability of the treated mice. The behavior study (training session) was carried out for four days. After the training session, a probe test was conducted by removing the hidden platform. In the probe trial, the number of crossings, the latency to the platform, and the time spent in the target quadrant were calculated. All the data were recorded by a video tracking system (SMART, Panlab Harvard Apparatus, Bioscience Company, Holliston, MA, USA).

### 4.4. Y-Maze Test

For the assessment of spatial working memory, Y-maze tests were performed, made of black painted plastic. The Y-maze test apparatus contains three arms, each arm of the maze was 20 cm high, 50 cm long, and 10 cm wide at both the bottom and top. The mice were placed at the center of the apparatus and were permitted to move freely in the apparatus for 8 min in each arm. The number of arm entries were observed visually. The spontaneous alternation was defined as the successive entry of mice in three arms in an overlapping set of triplets. The spontaneous alternation behavior percentage (%) was calculated using the following formula: successive triplet sets (entries in three different arms consecutively)/total number of arm entries-2) × 100. The higher percentage of spontaneous alternation behavior was considered to show an improved cognitive performance, and vice versa.

### 4.5. Homogenization of Mouse Brain

Homogenization of the mouse brain was performed as described previously [123,124]. After behavioral analysis, all the mice were anesthetized with ketamine/xylazine and then sacrificed. The brain tissues were removed immediately, and the cortex and hippocampus were carefully separated. The tissues were homogenized within PRO-PREPTM extraction solution (iNtRON Biotechnology, Burlington, NJ, USA) and an equal volume of 20–30 μg of protein was mixed with 2× Sample Buffer (Invitrogen, Waltham, MA, USA), then centrifuged at a speed of 13,000 rpm for 25 min at 4 °C. The supernatant was collected and stored at −80 °C.

### 4.6. ROS Assay

The ROS assay was conducted to evaluate the reactive oxygen species (ROS) level in the brain of the experimental mice groups (*n* = 6 mice/group). The assay was mainly based on the formation of 2′7′ dichlorofluorescein (DCF) from oxidation of 2′7′-dichlorodihydrofluorescein diacetate (DCFH-DA). Briefly, the brain homogenates of the normal and treated mice groups were diluted 1:20 in ice-cold Lock’s buffer to produce 2.5 mg tissue/500 μL as the final concentration. The final reaction mixture of 1 mL of the Lock’s buffer (pH ± 7.4), 0.2 mL of homogenates, and 10 mL of DCFH-DA (5 mM) were incubated for 15 min at room temperature to form fluorescent DCF from DCFH-DA. The conversion of 2′-7′dichlorofluorescin diacetate DCFH-DA to DCF was evaluated using spectrofluorimeter at an excitation wavelength of 484 nm and an emission wavelength of 530 nm. To measure the conversion of DCFH-DA to DCF in the absence of homogenate (background fluorescence), parallel blanks were used. The ROS levels were measured and expressed as relative pmol DCF/mg protein.

### 4.7. LPO Assay

A lipid peroxidation (LPO) assay was performed as previously described with some modifications [36]. Evaluation of LPO is important for the assessment of oxidative stress. The LPO marker free malondialdehyde (MDA), in the cortical and hippocampal protein lysates, was assessed using a thiobarbituric-acid-reactive substance (TBARS) assay kit (Bio Vision, Milpitas, CA, USA) according to the manufacturer’s instructions.

### 4.8. Antibodies and Reagents

The following antibodies were used in immunoblotting and immunofluorescence studies: anti-Iba-1, anti-GFAP, Nrf2, HO-1, anti-TNF-α, anti-IL-1β, anti-p-NF-κB, Cox2, anti-caspase-3, anti-cytochrome-C, anti-Bax, anti-Bcl2, and anti-β-actin. These antibodies were obtained from Santa Cruz Biotechnology (Dallas, TX, USA). Primary antibodies were diluted in 1× TBST (Tris-buffered saline plus Tween) (1:1000), while secondary conjugated anti-mouse horseradish peroxidase (HRP) and conjugated anti-rabbit HRP were diluted 1:10,000 in 1× TBST, all these were purchased from Promega, Madison, WI, USA. For immunofluorescence studies, the secondary fluorescent antibodies goat anti-mouse and goat anti-rabbit were used, and diluted in 1 × 100 phosphate-buffered saline (PBS).

### 4.9. Immunoblotting

Expression levels of proteins were quantified by using a Bradford assay (Bio-Rad Protein Assay kit, Bio-Rad Laboratories, Hercules, CA, USA) as described previously, with some modification [47,125,126]. The protein samples were electrophoresed on SDS-PAGE and then transferred to polyvinylidene difluoride membranes (PVDF) (Millipore, Burlington, MA, USA). A protein marker (GangNam-STAIN, iNtRON Biotechnology, CA, USA) was loaded parallel determine the molecular weight of the protein. All the membranes were blocked in 5% skimmed milk to reduce the nonspecific bindings, and then incubated overnight with the primary antibodies at 4 °C. The next day, the membranes were washed with 1xTBST (3 × 10 min) and blocked with horseradish-peroxidase-conjugated secondary antibodies, diluted in 1× TBST for 1 h, and then washed with 1× TBST (4 × 12 min). After washing, the membranes were developed in a dark room and the bands were detected using an enhanced chemiluminescent (ECL) detection reagent (EzWestLumiOne, ATTO, Tokyo, Japan). The immunoblots bands were obtained using X-ray films. For band quantification, ImageJ software (v. 1.50, NIH, Bethesda, MD, USA) was used and the graphs were evaluated using GraphPad Prism 6 software (San Diego, CA, USA).

### 4.10. Immunofluorescence Analysis

Immunofluorescence analysis was performed as described previously [127,128,129,130]. Briefly, the brain sections were dehydrated overnight and then rehydrated on next with 1% 1x phosphate-buffered saline (PBS) for 10 min. The slides were then treated with proteinase K for 5 min, then washed again with PBS and blocked with 5% normal serum for 1 h. After blocking, the slides were carefully incubated with primary antibodies (1:100) for 24 h at 4 °C. After incubation with primary antibodies, the slides were washed with 1% PBS and treated with fluorescein isothiocyanate (FITC)-labeled secondary antibodies for 90 min at room temperature. The slides were then washed and treated with 4′,6-diamidino-2-phenylindole (DAPI) for detection of the nucleus. The slides were covered using coverslips with a fluorescent mounting medium (FluoView FV 1000; Olympus, Tokyo, Japan) and the images were captured by a confocal scanning microscope (FV1000MPE). The captured results were evaluated by the relative integrated densities using ImageJ software (version 1.50, NIH, 1 March 2016; https://imagej.nih.gov/ij/, USA) and the graphs were created through PRISM6 software.

### 4.11. Statistical Analysis

The Western blot bands were scanned and analyzed using densitometry in the computer-based Sigma Gel System (SPSS Inc., Chicago, IL, USA). The immunofluorescence results were analyzed using ImageJ software, and the densities were calculated in arbitrary units. The data are presented as the mean ± standard error of mean (SEM). One-way analysis of variance (ANOVA) and Student’s t-test were used for comparison of the different groups. Statistical analyses were performed using Graph-Pad Prism 6 software. P values less than 0.05 were considered to show a significant difference between the groups; Western blots and graph bars are represented as Control, TBI (TBI-treated group), TBI + Lupeol (TBI and lupeol-treated group), and Sham (Lupeol-alone treatment group).

## 5. Conclusions

In summary, the findings of the current study demonstrated that lupeol is a potential neurotherapeutic candidate that markedly reversed TBI-induced glial cells activation, oxidative stress, neuroinflammation, apoptosis, and memory impairment in mouse brains. We propose that these underlying potent neuroprotective effects of lupeol against TBI-induced neurotoxicity might be due to the inhibition of glial cells activation and oxidative stress. Based on these findings, we suggest that lupeol is a safe, effective, and a promising neurotherapeutic agent. However, future studies are highly encouraged to further evaluate the underlying molecular mechanism and role of lupeol in neuroinflammation and in various age-related neurodegenerative disorders, particularly in AD.

## Data Availability

The authors hereby declares that the data presented in this study will be presented upon request from the corresponding author.
